# Syndemic pathways linking juvenile delinquency, structural vulnerability, and mental health: implications for exploitation risk

**DOI:** 10.3389/fpubh.2026.1858900

**Published:** 2026-06-15

**Authors:** Deirdre Colburn, Kimberly J. Mitchell, Bruce G. Taylor

**Affiliations:** 1 Crimes against Children Research Center, University of New Hampshire, Durham, NH, United States; 2 Public Health, NORC at the University of Chicago, Chicago, IL, United States

**Keywords:** exploitation risk, juvenile delinquency, mental health, social determinants of health, social support, structural vulnerability, syndemic, youth

## Abstract

**Introduction:**

Juvenile delinquency, mental health, social determinants of health, and social support are closely intertwined, yet the structural and relational processes linking them remain underexamined. Using a syndemic framework, this study examined whether structural vulnerability and social support were associated with the relationship between juvenile delinquency and depression and anxiety in a nationally representative sample of youth and young adults in the United States.

**Methods:**

After data-quality exclusions, the full survey sample included 5,311 respondents ages 10 to 34 years, and the present analyses were restricted to the 2,658 respondents assigned to the rotating module that included delinquency items. Survey-weighted descriptive analyses and hierarchical regression models were estimated, followed by exploratory parallel mediation analyses. About one-quarter of respondents reported any juvenile delinquency history.

**Results:**

Respondents with delinquency histories showed higher unadjusted depression and anxiety scores, greater adversity, greater social determinants of health burden, and lower social support. In adjusted models, however, the association between delinquency and depression and anxiety was substantially reduced after accounting for structural vulnerability and was further reduced and no longer statistically significant after social support was added. Both indirect associations were statistically significant in exploratory mediation models, and together they accounted for a substantial share of the observed relationship. A small subgroup who reported having been paid for sexual relations showed particularly high adversity and structural burden, although these findings were descriptive only.

**Discussion:**

These findings suggest that the mental health burden observed among youth with delinquency histories is embedded in broader social and structural disadvantage and may be more effectively addressed through interventions that reduce social determinants of health burden, such as material hardship, and strengthen social support.

## 1 Introduction

Youth who engage in delinquent behaviors, such as running away from home or foster care placements, are at higher risk for adverse outcomes including involvement in commercial sexual exploitation (CSE) victimization (1–5). Both juvenile delinquency and CSE involvement are associated with mental health issues including higher rates of depression, anxiety, and post-traumatic stress disorders (6–8). Recent research in the fields of psychology, sociology, epidemiology, and public health has shifted to examining upstream factors that affect mental health over and above more static factors like demographic predictors. Social and structural determinants of health (SDoH), or the conditions in which people are born, grow, live, work, and age, are modifiable factors that greatly influence health and well-being (9–11). How SDoH may affect the relationship between juvenile delinquency and mental health outcomes remains under explored.

Social support is likely to be central to this relationship. Youth involved in delinquent behavior often experience strained family relationships, weaker school connectedness, greater exposure to delinquent peer networks, and reduced access to supportive adults and caregivers, all of which are linked to greater behavioral risk and poorer adjustment (12–14). A lack of supportive relationships may worsen emotional distress on its own and may also make the effects of structural vulnerability harder to manage by leaving youth with fewer people to turn to when they are facing adversity. Prior research shows that family support, peer support, and broader social connectedness are linked to lower depressive symptoms and better adjustment among vulnerable adolescents, including those who have experienced adversity (15–17). For this paper, these findings suggest that structural vulnerability and social support are not just background factors. They are closely connected conditions that may help explain why delinquency is associated with poorer mental health.

Juvenile delinquency can involve a range of activities including running away, hitchhiking, begging or stealing, public intoxication, vandalism, arson, or gang activity—behaviors that may result in contact with the juvenile justice system and increase the risk of CSE involvement (1–5). Juvenile delinquency is unique from adult criminal behaviors in that (1) there may be greater potential for early intervention and system diversion (18) and (2) engaging in delinquency during childhood and adolescence can have long lasting consequences that persist into adulthood. Justice system contact can disrupt youths’ educational trajectories (19), limit future employment opportunities (20, 21), and contribute to future financial instability (22). Beyond educational and economic impacts, juvenile delinquency is associated with mental health concerns. The prevalence of mental health disorders is higher among those youth involved in the juvenile justice system when compared to community samples (23, 24) suggesting that mental and behavioral health concerns influence later delinquent behaviors. However, there is also evidence that supports increasing mental health concerns as a result of involvement in the juvenile justice system; research has found that youth in intake placements (i.e., places of initial contact with the justice system where youth are screened for system diversion or formal processing) had lower rates of suicidal behaviors and both internalizing and externalizing disorders compared to those with more advanced justice system involvement and delinquent histories (25). The relationship between juvenile delinquency and mental health outcomes is likely bidirectional. Understanding the factors that influence mental health concerns among youth with delinquent histories is crucial to informing screening, intervention, and treatment plans in juvenile justice settings.

Social determinants of health are associated with individual risk for juvenile delinquency and justice system involvement (26). In particular, neighborhood context, exposure to violence, and adversity or victimization experiences during childhood are contributing factors to delinquent behaviors among youth, while family structure and support acts as a protective determinant buffering the negative effects of community factors (26, 27). Social support may matter not only as a protective factor in its own right, but also as a mechanism linking delinquency to mental health. Youth with delinquency histories may experience weakened ties to caregivers, schools, and prosocial peers while also becoming more exposed to stressful or unstable environments. Those conditions can leave youth with fewer emotional and practical supports at the same time that their need for support is greatest. This makes social support conceptually distinct from, but closely intertwined with, broader structural disadvantage (13, 15, 16).

We examine these factors as potential explanatory pathways while recognizing the limits of cross-sectional data. Our aim is not to establish causal mediation, but to test whether structural vulnerability and social support each statistically account for part of the observed association between delinquency and mental health. We model them as parallel pathways because both are theoretically relevant, conceptually distinct, and likely to co-occur among youth with delinquency histories (13, 26), even though the cross-sectional design does not allow us to determine their temporal ordering (28, 29). A syndemic framework provides a useful lens for understanding the co-occurrence of and interactions between juvenile delinquency, mental health problems, and vulnerability to exploitation. Syndemics refer to the clustering and interaction of multiple adverse conditions, such as disease, poverty, trauma, and social marginalization, that are driven by structural inequities and reinforce one another to worsen health outcomes (30, 31). In this context, SDoH and deficits in social support may not only co-occur with delinquent behaviors but actively shape pathways through which delinquency becomes linked to both mental health burden and heightened vulnerability to commercial sexual exploitation. This framework is useful here because it directs attention to how behavioral risk, structural disadvantage, and social disconnection may cluster together and jointly intensify mental health burden, rather than focusing on each factor in isolation.

Despite growing recognition of the role of social determinants of health (SDoH) in shaping youth outcomes, limited research has examined how these structural and relational factors operate as mechanisms linking juvenile delinquency to mental health. Moreover, few studies have considered these relationships within a broader syndemic framework that accounts for the co-occurrence and interaction of behavioral risk, structural disadvantage, and social disconnection. Understanding these pathways is particularly important given their potential relevance for identifying youth at elevated risk for adverse outcomes, including vulnerability to exploitation.

The current study addresses these gaps by examining whether SDoH deficits and social support function as key pathways through which juvenile delinquency is associated with depression and anxiety among a nationally representative sample of youth and young adults. We include both youth and young adults to examine whether these relationships are present across a broader time period in the life course. Drawing on a syndemic framework, we conceptualize delinquency, structural disadvantage, and social support as interconnected processes that may jointly shape mental health outcomes. In doing so, we move beyond models that treat delinquency as an isolated behavioral risk factor and instead situate it within a broader system of social and structural conditions.

Specifically, this study addresses three research questions: (1) Is juvenile delinquency associated with higher levels of depression and anxiety? (2) To what extent are SDoH deficits and social support independently associated with mental health outcomes when accounting for delinquency and relevant covariates? (3) Do SDoH deficits and social support mediate the relationship between juvenile delinquency and depression and anxiety? Based on prior research, we hypothesized that delinquency would be associated with greater SDoH deficits and lower social support, and that these factors would, in turn, be associated with worse mental health outcomes. We further hypothesized that SDoH deficits and social support would account for a substantial portion of the association between delinquency and depression and anxiety.

In addition, we provide brief descriptive information on a subset of respondents reporting having been paid for sexual relations. We treat this item as a limited proxy indicator relevant to exploitation risk rather than as a comprehensive measure of commercial sexual exploitation. Although not the primary focus of the analysis, we include this brief examination of CSE to add to the overall literature on trafficking explored in this special issue. These descriptive findings offer context for understanding how the structural and relational vulnerabilities examined in this study may also relate to exploitation risk within a broader syndemic framework.

## 2 Materials and methods

### 2.1 Participants

Youth and young adults aged 10 to 34 years were invited to participate in the *Growing Up with Guns* (GWG) survey. There were four panel sources used to sample youth and young adults: (1) young adults age 18–34 from NORC’s AmeriSpeak Panel; (2) young adults age 18–34 from NORC’s Amplify AAPI Panel; (3) teens age 13–17 on the AmeriSpeak Teen Panel; and (4) preteens and teens age 10–17 living in AmeriSpeak Panel households who are too young to join the AmeriSpeak Teen Panel or had not otherwise joined AmeriSpeak.

### 2.2 Procedures

Data were collected for the three-wave longitudinal GWG survey from nationally representative panels of youth and young adults aged 10 to 34 years who participated in the National Opinion Research Center (NORC) AmeriSpeak panel and its’ sub-panels (32). AmeriSpeak is a national sample and is not limited to any specific state or region. AmeriSpeak is designed to cover approximately 97% of non-institutionalized U. S. households. This estimate refers to sampling-frame coverage and is achieved by supplementing the USPS Delivery Sequence File with additional in-person listing in selected areas. Households outside that frame include P. O. Box-only addresses, some addresses not listed in the USPS Delivery Sequence File, and some newly constructed dwellings. Households were initially recruited using USPS mailings, telephone, and incentives. A more detailed description of AmeriSpeak panel recruitment and overall methodology is published elsewhere (33). Because this survey included sensitive topics, parental consent for all youth and teens aged 10–17 was required. Parents were offered a $2 incentive for completion of the screener and consent survey. The study protocol was reviewed and approved by the University of Chicago Institutional Review Board. All adult participants provided informed consent. For respondents ages 10 to 17 years, parental permission and youth assent were obtained before participation.

AmeriSpeak adult panelists were asked if they were the parent or legal guardian for a preteen or teen aged 10–17 who could be invited to the survey. Parents were provided with information about the study and given the opportunity to provide consent for AmeriSpeak to contact their child(ren) living in the same household. If consent was obtained, AmeriSpeak invited all eligible preteens and teens in the household to participate. Adjustments were made during weighting to account for the fact that multiple youth per household could participate in the survey. Parental consent rate was 64.3% (32).

This study uses data collected for the Wave 1 GWG survey, which was fielded from September 2023 through January 2024 and was offered in English and Spanish via web and phone. Respondents received a cash equivalent of $20 for completing the survey. A total of 16,085 panelists were invited, of whom 5,461 completed the survey. Following standard data quality procedures, 150 cases were removed for either speeding through the survey, high refusal rates (i.e., skipping more than 50% of eligible questions), or straight-lining grid questions, resulting in a final analytic sample of 5,311 respondents.

To reduce respondent burden, the survey employed a planned missingness design in which all respondents completed a core set of items, and additional constructs were administered through randomly assigned rotating modules. Each respondent was assigned to complete two of four rotating modules. Measures of personal delinquency were included in one rotating module; therefore, the analytic sample for the present study was restricted to respondents who were randomly assigned to that module. This approach assumes data are missing at random due to random assignment of modules.

Survey weights were applied to produce nationally representative estimates. Weights began with panel weights (using the nominating parent’s weight for youth ages 10–17) and incorporated adjustments for parent screener completion, parental consent, the number of eligible youth in the household, and interview nonresponse. Final weights were calibrated to Current Population Survey benchmarks for age, sex, race/ethnicity, education, and Census Division.

### 2.3 Measures

#### 2.3.1 Delinquency

Juvenile delinquency was measured using 10 items adapted from the Rochester Youth Development Study Self-Report Delinquency scale (34). We asked whether, in the last 12 months (youth) or before age 18 (adults), respondents had engaged in each of the following behaviors: Run away from home, hitchhiked a ride with a stranger, begged for money or things from strangers, been drunk in public place, damaged, destroyed or marked up someone else’s property on purpose, set fire on purpose or tried to set fire to a house, building or car, tried to steal or actually stole money or things, been involved in gang or posse fights, been paid for having sexual relations with someone, made or tried to make someone have sex or engage in sexual activity with you. The 10-item sum score was highly skewed, showing a pronounced floor effect, as most participants did not endorse any delinquency behaviors, therefore a dichotomous yes/no measure was created to capture those who endorsed any of the items above vs. none.

#### 2.3.2 Social determinants of health

Social determinants of health (SDoH) were measured using self-report items across multiple domains including economic stability (not having enough money to pay bills and skipping meals because you did not have enough money for food), social context (10 items measuring adversity such as serious illness, accidents, family homelessness), health care (last time you saw a dentist for check-up, exam, teeth cleaning, or other dental work), and neighborhood and built environment (20 items measuring physical home conditions like mold, lead paint, not enough heat, or water leaks, and neighborhood conditions like presence of drugs, homelessness, graffiti). Using the sum of these items, we created a total SDoH deficit score and collapsed scores into four categories: 0 or 1 deficit, 2 deficits, 3 deficits, and 4 or more deficits. This index was intended to capture current structural and material conditions rather than lifetime adverse experiences. Although conceptually related, the SDoH measure and the separate adversity scale measure different domains, allowing us to distinguish ongoing contextual burden from earlier adverse experiences.

#### 2.3.3 Social support

Social support was measured using 7 items available in the survey with strong internal consistency (*α* = 0.92). Respondents were asked to think about the last time they were upset about something and rate how true each of the following items are: (1) “Someone was there for me when I was having a hard time”, (2) “Someone gave me a place where I could get away for a while”, (3) “Someone helped me get my mind off things”, (4) “Someone went with me to get some help”, (5) “Someone comforted me”, (6) “Someone stood up for me when I was in a tough spot”, (7) “Someone inspired me to work hard”. Responses ranged from Not true (1) to Mostly true (4). Scores across these items were summed to create a total social support scale (min = 7, max = 28, *M* = 20.11 (SD = 6.24)).

#### 2.3.4 Demographics

A series of questions collected participant demographic information including current age, sex assigned at birth (male, female, intersex), race, ethnicity, and sexual and gender identity. A dichotomous yes/no sexual gender minority variable created to capture participants who reported identifying with any sexual or gender minority identity was used in analyses.

#### 2.3.5 Adversity

Ten items were adapted from Turner and Butler (35) to measure non-violent lifetime adversities. These included living on the street or in shelter because you had no place to stay; your mother, father or guardian losing work and could not find a job; being sent or taken away from your family; parental incarceration; parental substance use; parental interpersonal violence; close family member death due to illness or accidental; parental divorce or separation; knowing someone who overdosed; and chronic medical condition. Responses (yes/no) were summed to create a total adversity score (*α* = 0.93) (min = 0, max = 10, *M* = 2.33 (SD = 2.23)).

#### 2.3.6 Depression and Anxiety

Depression and anxiety was measured using the Mental Health Inventory (MHI-5) (36), a five-item screening inventory of symptoms consistent with these clinical conditions. Response to these five items were summed to create a total depression and anxiety score (*α*  = 0.82) (min = 5, max = 30, *M* = 13.96 (SD = 4.92)).

### 2.4 Data analysis

Data were analyzed using StataNow/SE version 19.5. The “svy” prefix was used for all analyses to account for the complexity of the survey data and adjust for sampling weights that produce nationally representative estimates. To examine the bivariate relationship between juvenile delinquency and demographic factors, adversity, SDoH and social support, we used cross-tabulations with chi-square (for categorical variables) and compared group means any delinquency vs. none for continuous measures using survey-adjusted tests. Similarly, we compared mean levels of depression and anxiety scores for those with and without any self-reported juvenile delinquency. The threshold for statistical significance was set at *p* = 0.05. To examine whether juvenile delinquency was significantly associated with a change in depression and anxiety, we used hierarchical ordinary least squares (OLS) regression models adjusting for demographic and adversity covariates, subsequently adding SDoH deficit and social support scores into the model.

Finally, we conducted theory-informed exploratory mediation analyses using a parallel mediation framework to examine whether the association between juvenile delinquency and depression and anxiety operated indirectly through SDoH deficits and social support. Indirect effects were estimated within the survey-weighted framework. Because mediators and outcomes were measured cross-sectionally, mediation results should be interpreted as exploratory and not indicative of causal pathways. All analyses were conducted on the analytic subsample of respondents assigned to the rotating module containing delinquency measures as part of the survey’s planned missingness design.

## 3 Results

Table 1 provides an overview of sample characteristics. Our sample was comprised of 69.82% (*n* = 4,122) young adults (ages 18–34) and 30.18% youth (ages 10–17) (*n* = 1,189). Approximately half were assigned female at birth (47.39%) and 18.98% reported belonging to a sexual or gender minority identity group. The sample was nationally representative with respect to race and ethnicity.

**Table 1:** Sample characteristics.

Variable	Weighted % (se)
Age category	
Youth (*n* = 1,189)	30.18 (0.90)
Adult (*n* = 4,122)	69.82 (0.90)
Assigned Female at Birth (*n* = 3,174)	47.39 (0.93)
Sexual or Gender Minority (*n* = 1,076)	18.98 (0.70)
Race and Ethnicity	
American Indian (*n* = 227)	3.20 (0.30)
Asian (*n* = 682)	9.42 (0.46)
Black (*n* = 1,170)	16.85 (0.64)
Pacific Islander (*n* = 59)	1.02 (0.18)
Multiple race (*n* = 517)	7.46 (0.45)
White (*n* = 3,243)	69.15 (0.82)
Hispanic or Latino (*n* = 1,104)	22.16 (0.80)
Adversity total [mi*n* = 0, max = 10] (mean, se)	2.12 (0.04)
Delinquency	
Run away from home (*n* = 240)	7.73 (0.65)
Hitchhiked with stranger (*n* = 85)	3.07 (0.43)
Begged for money from strangers (*n* = 72)	2.72 (0.41)
Been drunk in public (*n* = 320)	10.40 (0.73)
Vandalized property (*n* = 210)	7.79 (0.67)
Set fire to house, building, car (*n* = 38)	1.34 (0.25)
Tried to steal or stole money or things (*n* = 329)	11.12 (0.82)
Involved in gang or posse fight (*n* = 40)	1.43 (0.31)
Been paid for sexual relations with someone (*n* = 48)	1.41 (0.24)
Made or try to make someone engage in sexual activity with you (*n* = 33)	1.03 (0.24)
Any (*n* = 731)	25.08 (1.12)
Social support score [mi*n* = 7, max = 28] (mean, se)	20.17 (0.12)
SDoH Deficit Count	
0 or 1 SDoH (*n* = 2,484)	48.9 (0.94)
2 SDoH (*n* = 1,381)	25.04 (0.81)
3 SDoH (*n* = 779)	14.26 (0.67)
4 or more SDoH (*n* = 667)	11.82 (0.60)
Depression and anxiety score [min = 5, max = 30] (mean, se)	13.75 (0.10)

About one-quarter (25.08%) reported any juvenile delinquency behaviors before the age of 18, including 1.41% (*n* = 48) who reported being paid for sexual relations with someone. Adult participants were more likely to report any juvenile delinquency experience (33.55%) compared to youth participants (5.80%), which likely reflects the longer period adults had to accumulate pre-age-18 delinquency experiences through retrospective report (Table 2). Juvenile delinquency was more likely among those identifying as a sexual or gender minority (39.10%) and those with American Indian (38.42%) or multi-racial (33.53%) identities. Those with any juvenile delinquency reported significantly higher mean adversity (3.40 vs. 1.73, *p* < 0.001) and depression and anxiety scores (15.71 vs. 13.07, p < 0.001), and lower social support (18.42 vs. 20.73, p < 0.001), compared to those with no juvenile delinquency history. Table 2 also shows the proportion of those in each demographic category, the mean social support and adversity scores, and the distribution of SDoH deficit scores among those who reported being paid for sexual relations with someone. Because cell sizes were notably small, statistical comparisons were not performed for this sub-sample.

**Table 2:** Juvenile delinquency history by demographic categories and adversity total, social support score, and depression and anxiety score among those with and without juvenile delinquency experience.

	CSE	Any vs. none		
	Been paid for sexual relations?^1^ (*n* = 48)	Any delinquency (*n* = 722)	No delinquency	*p*-value
	Row totals—weighted % (se)			
Sociodemographic characteristic				
Age category				<0.001
Youth (*n* = 1,189)	0.35 (0.24)	5.80 (1.13)	94.2 (1.13)	
Adult (*n* = 4,122)	1.87 (0.32)	33.49 (1.43)	66.51 (1.43)	
Assigned female at birth (*n* = 3,174)				0.5928
Female	2.10 (0.43)	24.47 (1.40)	75.53 (1.40)	
Not female	0.77 (0.23)	25.65 (1.71)	74.35 (1.71)	
Sexual or gender minority (*n* = 1,076)				<0.001
SGM	3.12 (0.75)	39.10 (2.78)	60.90 (2.78)	
Exclusively cisgender/heterosexual	0.96 (0.23)	21.46 (1.19)	78.54 (1.19)	
Race and ethnicity				
American Indian (*n* = 227)	2.49 (1.33)	38.42 (6.38)	61.58 (6.38)	<0.05
Asian (*n* = 682)	1.56 (0.86)	25.6 (2.76)	74.4 (2.76)	0.8488
Black (*n* = 1,170)	2.42 (0.80)	26.92 (2.49)	73.08 (2.49)	0.4195
Pacific Islander (*n* = 59)	3.77 (3.74)	27.04 (10.08)	72.96 (10.08)	0.8414
Multiple race (*n* = 517)	1.34 (0.62)	33.53 (4.01)	66.47 (4.01)	<0.05
White (*n* = 3,243)	1.15 (0.25)	24.29 (1.40)	75.71 (1.40)	0.2808
Hispanic or Latino (*n* = 1,104)	2.11 (0.70)	29.56 (2.84)	70.44 (2.84)	0.0533
Column mean (se)				
Adversity total	4.94 (0.41)	3.40 (0.11)	1.73 (0.06)	<0.001
Social support score	18.33 (0.86)	18.42 (0.32)	20.73 (0.19)	<0.001
Depression and anxiety score	17.54 (0.61)	15.71 (0.29)	13.07 (0.16)	<0.001
Column totals—weighted % (se)				
SDoH deficit total				<0.001
0 or 1 SDoH	15.53 (6.08)	28.31 (2.23)	56.66 (1.53)	
2 SDoH	12.25 (5.64)	25.07 (2.09)	23.22 (1.26)	
3 SDoH	14.23 (5.62)	23.49 (2.42)	12.64 (1.06)	
4 or more SDoH	57.98 (8.34)	23.13 (2.06)	7.48 (0.81)	

Chi-square comparisons between those with any vs. none delinquency.

^1^Cell sizes are <10 for many categories among those who reported being paid for sexual relations, therefore precluding statistical comparison. Column included to provide snapshot of CSE history among demographic categories.

### 3.1 Association between juvenile delinquency on Depression & Anxiety

Table 3 shows results of linear regression models of depression and anxiety score regressed on juvenile delinquency (Model 1), plus SDoH deficit score (Model 2), plus social support total (Model 3). All models control for age, sex at birth, SGM status, race, ethnicity, and total adversity score. After controlling for other sociodemographic predictors, juvenile delinquency was significantly and positively associated with an increase in depression and anxiety (*B* = 1.38, *p* < 0.001). With SDoH deficit score added into the model, the effect size of delinquency reduced to 0.89, but remained statistically significant (*p* < 0.01) in predicting depression and anxiety. SDoH score was independently associated with the outcome, with each increase in SDoH deficit score predicting greater increases in depression and anxiety. Social support score was added into the model in Model 3. With the addition of social support, the effect of delinquency on depression and anxiety was further reduced and became non-significant (*B* = 0.56, *p* = 0.0567). Social support was significantly (*p* < 0.001) and independently associated with the outcome, where every 1-point increase in social support score predicted a 0.22-point decrease in depression and anxiety score when holding all other predictors constant.

**Table 3:** Linear regression of depression and anxiety score regressed on delinquency, SDoH deficit count, and social support score (*n* = 2,658).

Predictor	Model 1	Model 2	Model 3
Delinquency (ref = no)			
*Yes*	1.38 (0.35) ***	0.89 (0.33) **	0.56 (0.30)
SDoH Deficit (ref = 0 or 1 SDoH)			
*2 SDoH*	–	1.01 (0.30) **	0.76 (0.30) *
*3 SDoH*	–	3.02 (0.43) ***	2.58 (0.40) ***
*4 SDoH*	–	2.84 (0.40) ***	2.25 (0.40) ***
Social Support total	–	–	−0.22 (0.02) ***

Models control for age, sex at birth, sexual or gender minority status, race, ethnicity, and total adversity.

*p < 0.05; **p < 0.01; ***p < 0.001.

A Spearman’s correlation was conducted to test for possible collinearity between SDoH and social support. The relationship was significant, but showed weak correlation (*r*_s_ = −0.20, *p* < 0.001). Additionally, sensitivity analyses were conducted stratifying by age group. In repeated models restricted first to only youth and then only adult participants, the outcome was the same: the effect size of delinquency was reduced but statistically significant with the addition of SDoH in the model and became non-significant with the inclusion of social support in the model. Therefore, we did not stratify by age for mediation analyses below.

### 3.2 Parallel mediation analyses

**Figure 1:**
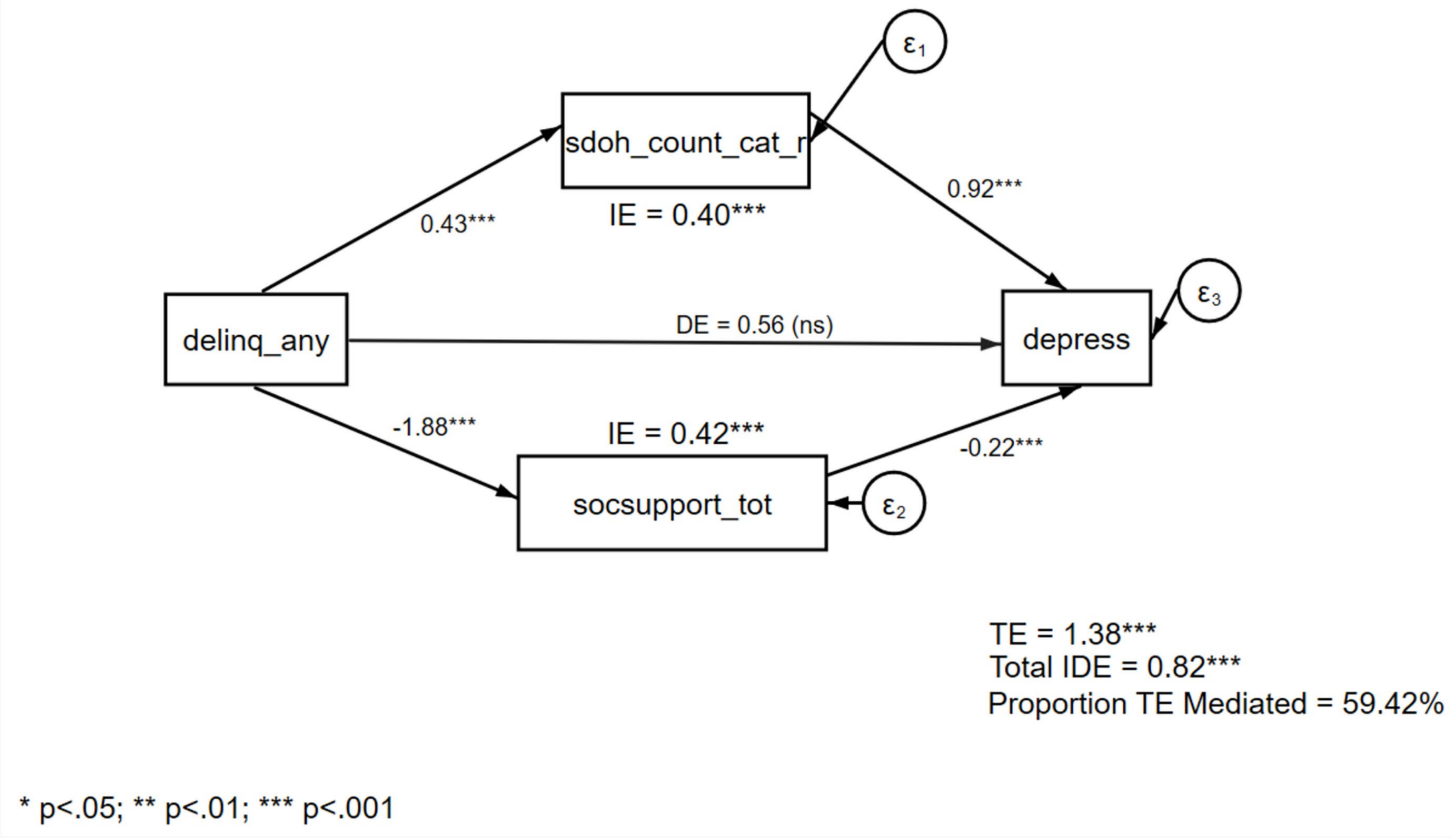
Path diagram showing exploratory parallel mediation analysis with SDoH and social support accounting for association between delinquency and depression outcome.

The reduction in effect size of delinquency on depression and anxiety score once SDoH and social support were added into the models prompted us to examine whether these factors statistically accounted for part of the observed association between delinquency and depression and anxiety. Table 4 shows the results of a formal parallel mediation analysis. In the parallel mediation model, both indirect associations were statistically significant. With SDoH, the indirect effect was 0.40 (*p* < 0.001), accounting for 28.98% of the total association between delinquency (TE = 1.38) with depression and anxiety. With social support, the indirect effect was 0.42 (*p* < 0.001), accounting for 30.43% of the total association between delinquency and depression and anxiety. Including both SDoH and social support in the exploratory mediation analysis simultaneously (i.e., parallel mediation), the indirect effect was 0.82 (p < 0.001), accounting for nearly 60% of the total association between delinquency and depression and anxiety. The contrast of SDoH and social support mediators was not significant, meaning that one relationship was not stronger than the other in this model. After accounting for both SDoH and social support mediators, the direct association (DE = 0.56) of delinquency with depression and anxiety was non-significant. Given the cross-sectional design, these findings should be interpreted as evidence that structural vulnerability and social support statistically account for a substantial portion of the observed association, rather than as confirmation of causal mediation. Figure 1 shows the path diagram for the mediation model.

**Table 4:** Exploratory parallel mediation analysis showing indirect association of delinquency after accounting for SDoH, social support, and both SDoH and social support.

Effect	Effect size	
Total effect	1.38 (0.35)***	–
Direct effect	0.56 (0.31)	–
Mediator	Indirect association (Bootstrapped SE)	Proportion association (TE) accounted for
*SDoH*	0.40 (0.08)***	28.98%
*Social support*	0.42 (0.10)***	30.43%
*SDoH + Social support*	0.82 (0.14)***	59.42%
Contrast of mediators	−0.02 (0.12)	–

***p < 0.001.

## 4 Discussion

The present study examined the relationship between juvenile delinquency and mental health outcomes within a framework that considered SDoH and social support as potential explanatory factors. Juvenile delinquency was initially associated with higher depression and anxiety scores, but this association was substantially reduced after accounting for greater SDoH burden and lower social support. These findings suggest that the mental health burden observed among youth with delinquency histories may be closely tied to broader structural and relational conditions, rather than to delinquency alone as an isolated behavioral marker.

These findings align with a syndemic framework, which emphasizes the co-occurrence and interaction of multiple adverse conditions that are rooted in structural inequities and that reinforce one another to worsen health outcomes (30, 31). In this study, juvenile delinquency, structural disadvantage, and diminished social support can be understood as interconnected components of a broader system of vulnerability. Rather than operating independently, these factors appear to cluster and interact in ways that amplify risk for poor mental health. This interpretation shifts the focus from individual behavior toward the social and structural context in which that behavior is embedded.

Several mechanisms may explain the observed association between delinquency and increased SDoH deficits. Youth who engage in delinquent behaviors are more likely to come into contact with the juvenile justice system (36), which can disrupt educational trajectories (19), limit future employment opportunities (20, 21), and contribute to financial instability (22). Labeling and stigma associated with delinquency (37, 38) may also lead to exclusion from institutions such as schools and workplaces, further entrenching structural disadvantage. These processes reflect broader patterns of institutional marginalization that can exacerbate existing inequities and contribute to the accumulation of SDoH-related risks over time.

In parallel, delinquency may also contribute to reductions in social support through multiple pathways. First, scholars have long theorized that involvement in delinquent behavior is often associated with increased affiliation with similarly marginalized or deviant peer groups (39), which may not provide the same level of emotional or instrumental support as prosocial networks. At the same time, relationships with family members, educators, and other supportive adults may become strained or disrupted (15, 27). Contact with the juvenile justice system may further weaken ties to supportive institutions and individuals. Together, these dynamics suggest that social support is not simply absent among delinquent youth but may be actively eroded through social and institutional processes.

Importantly, social support emerged as a strong and independent factor associated with lower depression and anxiety and accounted for a substantial portion of the indirect association linking delinquency to mental health outcomes. This finding highlights the critical role of supportive relationships as a potential buffer within this system of risk. Within a syndemic framework, social support may represent a key leverage point for intervention, with the potential to mitigate the effects of both structural disadvantage and behavioral risk.

Although only a small proportion of respondents reported having been paid for sexual relations, the descriptive patterns for this subgroup are notable. These respondents showed especially high adversity and structural burden, which is consistent with the broader literature showing that exploitation-related experiences often emerge in contexts marked by cumulative disadvantage, social disconnection, and prior victimization (40). From a delinquency perspective, behaviors such as running away, survival-based offending, public intoxication, and street involvement may signal exposure to the same structural and relational conditions that heighten vulnerability to exploitation. In that sense, delinquency may sometimes function less as an isolated behavioral problem than as an indicator of broader instability and unmet need.

These patterns can also be interpreted through the lens of structural violence, in which social, economic, and institutional systems systematically disadvantage certain groups and limit access to resources and opportunities (41, 42). Institutional responses to delinquency, including punitive justice system involvement and social labeling (37), may inadvertently reinforce cycles of marginalization by restricting access to education, employment, and supportive networks. In this way, structural conditions not only shape the emergence of delinquent behaviors but also influence the downstream consequences for mental health and exploitation risk.

Future research should test these relationships longitudinally, distinguish self-reported delinquency from formal justice-system contact, and examine whether structural vulnerability and social support operate differently across developmental stages and subgroups. Work that integrates richer measures of exploitation-related experiences would also clarify whether delinquency-related indicators improve early identification of youth facing elevated exploitation risk.

### 4.1 Limitations

Several limitations should be considered when interpreting these findings. First, because the data were highly skewed, our delinquency measure was collapsed into a binary any/none item rather than included as a sum score or categorical variable. It is possible that separate types of delinquency behaviors uniquely interact with social factors to differentially predict mental health outcomes. Future research examining these relationships should consider including delinquency as a categorical or cumulative sum score measure. The cross-sectional measurement of SDoH and social support limits the ability to establish temporal ordering, and it is possible that these relationships are reciprocal rather than strictly unidirectional. Moreover, though our mental health measure is current, we cannot establish definite causal ordering between constructs. It is also possible, and likely, that historical adversity and current SDoH are overlapping phenomenon and additional research is needed to parse out this relationship. In addition, the youth and adult subsamples differ not only in age, but also in the reporting frame for delinquency, with youth reporting on the past 12 months and adults reporting retrospectively on pre-age-18 experiences. This limits direct comparability across age groups. Therefore, the observed associations may reflect differences in developmental stage, recall, or exposure period. For adult participants, retrospective reporting of delinquency introduces additional uncertainty, as current mental health and social conditions may be influenced by unmeasured factors occurring after adolescence. The small number of respondents reporting transactional sexual experiences precluded statistical comparisons for this subgroup, and all measures relied on self-report, which may be subject to recall or social desirability bias. Despite these limitations, the consistency of findings across multiple analytic approaches supports the robustness of the observed relationships.

### 4.2 Implications

From a public health perspective, these findings underscore the importance of multi-level interventions that extend beyond individual behavior change. Youth with delinquency histories may benefit from responses that address both behavioral risk and the broader conditions in which that risk is embedded. Addressing SDoH, such as housing instability, economic insecurity, and access to healthcare, may be critical for improving mental health outcomes among youth with delinquent histories. In practice, screening approaches in juvenile justice, schools, child welfare, and community-based settings may be strengthened by assessing not only delinquent behavior, but also these broader indicators of structural burden and the availability of supportive adults. At the same time, interventions that strengthen social support networks, including family-based approaches, mentorship programs, and community connections, may help buffer against the compounding effects of structural disadvantage.

The findings also suggest that interventions focused only on behavior control are likely to miss a substantial part of the problem. For the juvenile justice system specifically, these findings suggest that screening should be linked to concrete referral and follow-up processes rather than functioning as a stand-alone intake exercise. They also support trauma-informed and family-engaged responses that do not rely solely on surveillance or sanctions, and they point to the value of diversion and reentry strategies that connect youth with behavioral health care and community-based supports when structural burden and social disconnection are high (43). The juvenile justice systems needs to recognize the role of adversity, structural burden, and social disconnection in shaping youths’ needs, as well as reentry planning that prioritizes continuity of mental health care and connection to community-based supports (43).

Finally, efforts to prevent commercial sexual exploitation may similarly benefit from early identification of youth experiencing high SDoH burden and social disconnection. The descriptive patterns observed among respondents reporting having been paid for sexual relations further suggest that overlapping indicators of delinquency, structural burden, and reduced social support may help identify youth who face elevated vulnerability to exploitation and who may benefit from earlier, more integrated support.

## 5 Conclusion

This study suggests that the relationship between juvenile delinquency and mental health is best understood within a broader system of intersecting structural and social factors. SDoH deficits and diminished social support appear to be central mechanisms through which delinquency is linked to depression and anxiety and may also contribute to heightened vulnerability to exploitation. Addressing these interconnected conditions may be essential for improving outcomes among at-risk youth and for disrupting pathways within a broader syndemic of mental health burden, poverty, and exploitation.

## Data Availability

Individual participant data that underlie the results reported in this article, after de-identification, will be available December 31, 2026 to investigators whose proposed use of the data has been approved by the project leads, have IRB approval and approved by an independent review committee identified for this purpose. Proposals should be sent directly to either Bruce Taylor (taylor-bruce@norc.org) or Kimberly Mitchell (kimberly.mitchell@unh.edu) for approval and to gain access. Data requesters will also need to sign a data use agreement. Twelve months after project completion, the data will be available in the Open Science Framework (https://osf.io/) but without investigator support other than deposited metadata.
